# Using lifestyle interventions and the gut microbiota to improve PTSD symptoms

**DOI:** 10.3389/fnins.2024.1488841

**Published:** 2024-12-03

**Authors:** Steven G. Sugden, Gia Merlo

**Affiliations:** ^1^Department of Psychiatry, Spencer Fox Eccles School of Medicine, University of Utah, Salt Lake City, UT, United States; ^2^Department of Psychiatry, NYU Grossman School of Medicine, New York, NY, United States

**Keywords:** brain gut microbiota system, neuroplasticity, mental health, posttraumatic stress disorder, PTSD, window of tolerance, lifestyle psychiatry, neuroinflammation

## Abstract

Posttraumatic stress disorder is part of a spectrum of psychological symptoms that are frequently linked with a single defining traumatic experience. Symptoms can vary over the lifespan in intensity based on additional life stressors, individual stability, and connectedness to purpose. Historically, treatment has centered on psychotropic agents and individual and group therapy to increase the individual’s window of tolerance, improve emotional dysregulation, and strengthen relationships. Unfortunately, there is a growing segment of individuals with posttraumatic stress disorder who do not respond to these traditional treatments, perhaps because they do not address the multidirectional relationships between chronic cortisol, changes in the brain gut microbiota system, neuroinflammation, and posttraumatic symptoms. We will review the literature and explain how trauma impacts the neuroendocrine and neuroimmunology within the brain, how these processes influence the brain gut microbiota system, and provide a mechanism for the development of posttraumatic stress disorder symptoms. Finally, we will show how the lifestyle psychiatry model provides symptom amelioration.

## 1 Introduction

The importance of the brain gut microbiota system (BGM) in mental health is being progressively recognized as a linking factor in mental and brain health. The multidirectional relationship between the brain, gut, and gut microbiota along the BGM system affects nutrient absorption and utilization. It substantially influences cognitive processes, mood regulation, neuroplasticity, and other mental and brain health indices (Merlo et al., 2024). Similarly, many chronic mental illnesses that increase the inflammatory process may promote dysbiosis, which impairs the gut microbiota function and nutrient absorption and, in turn, further worsens mental and brain health (Capuco et al., 2020; Berding et al., 2021). Emerging evidence links posttraumatic stress disorder symptoms to gut dysbiosis.

Posttraumatic stress disorder (PTSD) is part of a spectrum of psychological symptoms that is frequently linked with a single defining traumatic experience. More precisely, trauma is a complex phenomenon that manifests in varying degrees of intensity and impairment for individuals and communities who experience it. Symptoms can vary over the lifespan in intensity based on additional life stressors, individual stability, and connectedness to purpose. Particularly vulnerable populations that are at risk of developing pervasive PTSD symptoms include children (Benjet et al., 2016), healthcare workers (Sun et al., 2021), people of color, veterans (McClendon et al., 2020), and those who experience mental health (Howgego et al., 2005), housing insecurity (Ayano et al., 2020), poverty (Mayo et al., 2022), and substance use (Najavits et al., 2020).

Individuals with PTSD experience higher rates of medical comorbidities, including metabolic syndrome, obesity, hypertension, Type II diabetes Mellitus, and cardiovascular disease. They also report higher rates of substance use (Cottler et al., 1992), which leads to more complicated treatment outcomes (Flanagan et al., 2016). Additionally, 20 to 30% of non-veterans with PTSD report comorbid chronic pain whereas 49–80% of U.S. Veterans with PTSD report chronic pain. Both populations are significantly higher than the reported general prevalence of chronic pain, 12% in women and 6% in men (Fishbain et al., 2017). Of note, individuals with PTSD and chronic pain have an odds ratio of 4.79 (95% Cl 1.81–12.69) of ever attempting suicide (Reed 2nd et al., 2024). Using 2018 data, experts calculated the total excess economic burden of PTSD in the U.S. was estimated at $232.2 billion annually ($19,630 annually per individual with PTSD) (Davis et al., 2022). As such, many experts declare trauma and the development of PTSD as a public health priority (Watson, 2019; Magruder et al., 2017).

Historically, treatment has centered on psychotropic agents and individual and group therapy (Thakur et al., 2022; Bandelow et al., 2023) to increase the individual’s “window of tolerance” (Corrigan et al., 2011), improve emotional dysregulation (van der Kolk et al., 1994), and strengthen relationships (Long, 2022). Unfortunately, there is a growing segment of individuals with PTSD who do not respond to these traditional treatments (Bisson et al., 2020). The rates of suicide are also higher for those with PTSD than for those with mental illness or the general public (Chou et al., 2020). We will review the literature and explain how trauma impacts the neuroendocrine and neuroimmunology within the brain, how these processes influence the GMB system, and provide a mechanism for the development of PTSD symptoms. Finally, we will show how the lifestyle psychiatry model provides symptom amelioration.

## 2 PTSD and other symptomatic traumas

The Diagnostic and Statistic Manual of Mental Illness, fifth edition, text revision (DSM-5-TR) identifies the central feature of PTSD as the exposure to an actual death, serious injury, or sexual violence either directly, through witnessing, or through learning about the event. These events may be caused by nature (e.g., natural disasters) or caused by others (e.g., accidents, catastrophes, intentional acts, etc.) (TIP 57, 2024). The diagnostic challenge has centered on classifying what constitutes an exposure, whether it can be a series of exposures, and how it impacts neuroplasticity and the development of symptoms (Condon et al., 2023).

### 2.1 Impact on the brain gut microbiota system

There are growing lines of evidence connecting the BGM system and PTSD-related symptoms. Gut dysbiosis develops when there is an unhealthy change in gut bacteria, which alters gut health function. Meta-analyses have shown significantly lower concentrations of healthy microbes (i.e., *Actinobacteria, Lentisphaerae*, and *Verrucomicrobia*) (Petakh et al., 2024; Hemmings et al., 2017) and higher concentrations in unhealthy microbes (i.e., *Enterococcus, Escherichia*, and *Shigella*) (Hemmings et al., 2017) in individuals with PTSD. These changes may be the result of chronic stress and response to cortisol (Tetel et al., 2018) and stress hormones or inflammatory pathways, or the changes may occur due to the activation of pre-existing epigenetic factors within the gut (He et al., 2024). Additionally, individuals with significant dysbiosis experience more severe traumatic symptomatology (Hemmings et al., 2017).

#### 2.1.1 Neuroinflammation pathway

Dysbiosis of the gut microbiota leads to further dysregulation within the neuroinflammation or neuroendocrine pathways, which in turn impacts mental health domains (Merlo et al., 2024). The growth of *Enterobacteriaceae*, especially *Escherichia, Shigella, Proteus*, and *Klebsiella*, can increase gut levels of enterotoxin (Dicks, 2022). Additionally, they can release lipopolysaccharide (LPS) from their own cells, which may impair gut-associated lymphoid tissue (GALT), which includes the multi-follicular Peyer’s patches of the ileum, the numerous isolated lymphoid follicles (ILF) distributed along the length of the intestine, and the vermiform appendix (Mörbe et al., 2021). LPS also increases blood–brain barrier permeability to inflammatory markers, altering the microglia of the CNS as it promotes gliosis, neuronal damage (Yu L. W. et al., 2022), and depletion of neurotrophic growth factors like brain-derived neurotrophic factor (BDNF) (Calcia et al., 2016).

#### 2.1.2 Neuroendocrine pathway

Furthermore, LPS has been shown to activate the hypothalamic pituitary adrenal axis (HPA) (Farzi et al., 2018). There is a growing body of literature showing how gut microbiota affects neurotransmitters that function as hormones. Serotonin is derived from the essential amino acid tryptophan, which gets absorbed within the kynurenine pathway (Horn et al., 2022) and regulated within the gut (Barber et al., 2021; Rusch et al., 2023). *Enterobacteriaceae* are histamine-producing bacteria (Mou et al., 2021), and excess histamine is linked with visceral gut hypersensitivity, increased gut permeability, and altered gut motility (Vanuytsel et al., 2023). *Enterobacteriaceae* also promotes the conversion of dopamine from tyrosine, making it more abundant for dopamine-mediated networks, like the reward pathway (Hamamah et al., 2022). Additionally, individuals with dysbiosis struggle to ferment complex carbohydrates into short-chain fatty acids (SCFAs) that are able to downregulate the production of dopamine (Hamamah et al., 2022).

### 2.2 Development/neuropathology

Central to developing neuropathology sequela is activating the individual’s sympathetic nervous system via the HPA axis. In a non-stress environment, cortisol is released by the adrenal glands within a predictable circadian rhythm, typically with spikes at the time of arousal (Dedovic et al., 2009). The low-dose cortisol aids in recovery and daily repair within the cerebrum. Cortisol is self-regulated via a negative feedback loop within the central nervous system, particularly the HPA. Within the limbic system, the amygdala (AG) and, to a lesser degree, the hippocampus (HC) and medial prefrontal cortex (mPFC) constantly monitor the individual’s environment in a process known as neuroception. If a threat or a perceived threat is detected, the limbic system activates the HPA, and cortisol, epinephrine, and norepinephrine are released via the sympathetic response, which enables the individual to “fight or flight” as needed. The sympathetic activation allows for increased focus and increased reflexive behaviors (Dedovic et al., 2009). The acute sympathetic response also inhibits the release of dopamine from the nucleus accumbens (NA) (Baik, 2020). Once the threat has passed, the HPA down-regulates the stress response, and the individual returns to their baseline (Dedovic et al., 2009). Neuropeptide Y (NPY) may play a role in downregulating norepinephrine within this response (Scioli-Salter et al., 2015).

In situations of significant trauma, complex trauma, and recurring or reactivating triggering trauma, the HPA is unable to down-regulate the acute release of cortisol. The heightened epinephrine and norepinephrine levels within the prefrontal cortex (PFC) reduce cognitive abilities (Arnsten, 2015) and may be linked with PFC atrophy in veteran populations with persistent symptomatology (Cardenas et al., 2011). Interestingly, NPY baseline levels are lower in individuals with chronic trauma (Scioli-Salter et al., 2015). Additionally, HC volume is inversely related to chronic cortisol exposure, which may explain further memory difficulties, especially as longitudinal studies within veteran populations showed a decline in facial recognition (Samuelson et al., 2009) and verbal ability (Cardenas et al., 2011).

Heightened cortisol also leads to heightened AG activations, which may heighten the neuroception role of the AG, explaining the hyper-vigilant symptoms frequently associated with PTSD (Dedovic et al., 2009). Finally, an additional aspect of the chronic trauma response is its activation of the parasympathetic nervous system (Porges, 2021), which contributes to the “freeze” or “fawning” symptoms that are frequently experienced. In 1999, Psychiatrist Daniel Spiegel coined the phrase “window of tolerance” to describe the zone of euthymia between the sympathetic and parasympathetic states and postulated how symptomatic relief from PTSD would be achieved by further opening the window of tolerance zone (Siegel, 1999).

Another aspect of the trauma response is the activation of the inflammatory response, as seen in the elevated levels of c-reactive protein, interferon-gamma, interleukin 6, interleukin 1 beta, and tumor necrosis factor-alpha. These pro-inflammatory proteins are able to pass through the blood–brain-barrier, impact glial cells, and initiate the neuroinflammatory response through the activation of cytokines and prostaglandins (Yirmiya and Goshen, 2011). Chronic elevated cortisol also impacts the NA. Instead of suppressing dopamine, which happens in the acute response, the NA responds to chronic cortisol by stimulating the release of dopamine (Baik, 2020). Apart from hijacking the reward pathway (Volkow et al., 2019), chronic dopamine release further promotes neuroinflammation (Sugden et al., 2024a).

NPY has become a potential target of interest. When present, NPY has been shown to reduce amygdala reactivity to pain by decreasing the emotional and behavioral associated responses (Scioli-Salter et al., 2015). Similarly, lower concentrations of NPY have been associated with worsening PTSD-related symptoms (Scioli et al., 2020). The dorsal anterior cingulate cortex (dACC) is another circuit of interest as it has been shown to be integral in processing both physical and social pain (Eisenberger, 2012). The dACC provides sensory input to the amygdala and plays an important role in emotional salience (Selemon et al., 2019). Individuals with PTSD have heightened activity in the dACC and along the pathways connecting to the amygdala (Selemon et al., 2019).

### 2.3 Development of symptoms

This section provides an overview of the processes of memories and their relationship to symptom formation, highlighting pertinent definitions. Explicit memory is the conscious recollection of facts or experiences. Working or procedural memory centers on the ability to carry out tasks in a certain predictable manner. Implicit memory is a form of long-term memory that allows an individual to perform a task without recalling the experience.

Van Der Kolk et al. have proposed that, as a result of repeated traumatic events, the hippocampus fails to process the traumatic event as an explicit memory and instead records it as an implicit memory (Van Der Kolk et al., 1997). Additionally, Leuthi et al. evaluated healthy controls’ ability to process memory and noted that negative stimuli were highly disruptive for working memory processing (Luethi et al., 2009). As mentioned, one of the roles of the AG is neuroception, which detects threats or fear-promoting activities (Siegel, 1999) and re-activates the trauma pathway once the threat is detected (Sasmita et al., 2018). In a classic conditioning response, individuals react to these fear-perceiving moments, oftentimes unaware of their reaction, which is centered in their implicit memory. The patterns protect the individual from encountering the same fear response (LeDoux, 2000).

As a result, individuals experience the trifecta of abnormal learning conditions, affective dysregulation, and altered cognitive cues of social circumstances via classically conditioned responses (Dedovic et al., 2009; Herman, 2008). Thus, flashbacks and hypervigilance keep the individual on guard; depression, despair, and hopelessness help the individual to be seen but not heard; fear restricts relationships and the freedom to act; and shame pushes the individual into invisibility (Fisher, 2021). Each symptom represents how the brain and body adapt to a chronic threat condition (van der Kolk et al., 1996).

## 3 Lifestyle interventions

Lifestyle psychiatry provides a unique, evidence-based lens for the treatment of mental health disorders like PTSD. Lifestyle psychiatry organizes evidence into six pillars: nutrition, physical activity, restorative sleep, stress management, connectedness, and avoidance of toxic exposures. These pillars can further be modified by social determinants of health and individual personality factors (not to be confused with personality disorders) (Merlo and Fagundes, 2023). Many medical societies have adopted lifestyle interventions as first-line and/or adjunct for chronic medical conditions such as diabetes, hypertension, heart disease, and cancer due to their effectiveness in ameliorating the effects of chronic inflammation (Parkinson et al., 2023), which is also a significant pathway in mental illness, particularly PTSD (Lee et al., 2022). Additionally, lifestyle interventions promote positive neuroplasticity (Sugden et al., 2024a), strengthen the BGM system (Campaniello et al., 2022), decrease the impacts of neuroinflammation, and increase the window of tolerance.

### 3.1 Nutrition

After reviewing the Nurses’ Health Study II Data from 51,965 women, Kim et al. noted that individuals with mild to moderate and severe PTSD symptoms did not show improvement in their diet quality over 20 years as measured by Alternative Healthy Eating Index-2010 compared to women who did not experience PTSD symptoms. Interestingly, those who experienced trauma during the 20-year study were more likely to adopt the eating patterns of the severe symptom PTSD group (Kim et al., 2021). Additionally, the authors noted that individuals with PTSD consumed less amounts of healthy flavonoids (Kim et al., 2021). In a systematic review by van den Berk-Clark et al., the authors compiled 19 studies of over 1.6 million participants and noted a higher rate of obesity within the population from veteran and population samples with PTSD compared to individuals who were not diagnosed with PTSD. Additionally, they calculated an odds ratio of 1.25 (95% CL: 1.20–1.30), showing significant patterns in food consumption between the two groups, with those diagnosed with PTSD most likely to consume fast food (van den Berk-Clark et al., 2018). Fast food, as well as other ultra-processed foods, increase the inflammatory process within the gut (Tristan Asensi et al., 2023) and may promote neuroinflammation as well (Firth et al., 2019).

A key component of ultra-process-rich food is its lack of dietary fiber. Diets rich in dietary fibers promote certain types of healthy bacteria (i.e., *Bifidobacterium, Lactobacillus, Lachnospiraceae, Blautia, Coprococcus, Roseburia*, and *Faecalibacterium*), which are able to break down complex carbohydrates into SCFA, via fermentation (So et al., 2018). The SCFAs (e.g., acetate, propionate, and butyrate) (Berding et al., 2021) have a wide range of host activities, including metabolism, cell differentiation, gene regulation (Berding et al., 2021; Martin-Gallausiaux et al., 2021), and regulating anti-inflammatory and pro-inflammatory cytokines (Maslowski et al., 2009). Within the gut, SCFAs strengthen the epithelial barrier functions, maintain an environment favorable for commensal bacteria, and inhibit pathogen growth (Martin-Gallausiaux et al., 2021).

The most robust data shows an improvement in mental health symptoms with adopting a whole-food, plant-based diet, which contains dietary fiber. Jacka et al. were one of the first to conduct a randomized control study in which patients were encouraged to limit their consumption of ultra-processed food. Not only did participants show an overall improvement in depression symptoms, the authors calculated the number needed to treat (NNT) was 4.1 (Jacka et al., 2017). The authors also revealed that the whole-food, plant-based diet was more cost-effective than a typical diet for participants (Chatterton et al., 2018). There have been two recent feasibility studies showing an improvement in PTSD symptoms. Herbert et al. identified ten U.S. veterans with PTSD and chronic pain who were given 2-weeks of plant-based meals high in dietary fiber, followed by 2-weeks of a regular diet. The veterans reported improvement in both chronic pain and PTSD symptoms (Herbert et al., 2023). Arcan et al. studied responders from the World Trade Center disaster. Responders either received nutritional counseling or help to adopt a Mediterranean diet. Those with the Mediterranean diet showed a greater change in the Posttraumatic Check List-DSM 5 (PCL-5) (Arcan et al., 2023).

### 3.2 Physical activity

There is less data regarding the amount of exercise in individuals with PTSD. Data from the U.S. Department and Human Services indicate that 20–23% of men and 16–20% of women meet the national standards of cardiac exercise (150 min of light to moderate/week) and weight training (2 sessions/week) (U.S. Department of Health & Human Services, 2018). Review articles that look at the benefits of exercise infer that the amount of exercise in the PTSD population is less than that of the average population (van den Berk-Clark et al., 2018; Hall et al., 2015).

Multiple reviews and meta-analyses have started examining the benefits of exercise and the kind of exercise that improves PTSD-related symptoms. Rosenbaum et al. conducted a meta-analysis of non-veteran subjects engaging in combined physical activity showed a significant improvement in their PTSD symptoms (hedges *g* = −0.35, 95% CI: −0.63 to −0.07, *p* = 0.02) (Rosenbaum et al., 2015). Similarly, Whitworth and Ciccolo focused on 13 studies of U.S. Veterans that combined multiple forms of exercise (aerobic, stretching (e.g., yoga)) and noted that PTSD symptoms were inversely related to the amount of total exercise time (Whitworth and Ciccolo, 2016). Bryant et al. randomized brief aerobic exercise to PTSD therapy and reported an improvement in CAP-2 scores 6 months later (Bryant et al., 2023).

Yu et al. showed in their meta-analysis of 11 studies of 573 individuals with PTSD that PTSD-related symptoms improved significantly with yoga (stretching exercises) and multi-modal exercise (the combination of aerobic and stretching and/or resistant training) (Yu Q. et al., 2022). Jadhakhad et al. evaluated 13 studies from 4 countries involving 531 patients and showed the patients had the most significant improvement in their symptoms when they exercised between 30 and 60 min, three times a day, in multi-modal exercises (Jadhakhan et al., 2022). Zaccari et al. showed a decrease in cortisol saliva following a 10-week yoga training session in a pilot study with 27 veterans (Zaccari et al., 2020). Aerobic exercise increases the release of neurotrophic factors (i.e., BDNF and serotonin), improves mitochondrial energy utilization, and decreases neuroinflammation by modulating microglial activity and by reducing the release of adipose IL-6 (Sugden et al., 2024a). Cardiorespiratory fitness has also been shown to elevate plasma NPY levels (Scioli-Salter et al., 2016), decrease chronic pain, and improve affective dysregulation (Scioli et al., 2020).

Although not specific to individuals with PTSD, physical activity has been shown to change the composition from unhealthy bacteria (i.e., *Bacteroides*) that lead to dysbiosis to *Roseburia, Lachnospira, Lachnospriaceae, Clostridiales*, and *Faecalibacterium*. These bacteria absorb fiber, produce SCFAs, and improve cardiometabolic fitness (Aya et al., 2021).

### 3.3 Restorative sleep

A consistent feature of PTSD is poor sleep, frequently made worse by the presence of nightmares (Shalev et al., 2017). There is also a correlation between the U.S. Veteran population with PTSD and the development of obstructive sleep apnea (Colvonen et al., 2015). Interestingly, U.S. Veterans who rated their sleep as poor to fair over 7 years were 60% more likely to develop the diagnosis of PTSD (Colvonen et al., 2015). Likewise, individuals with obstructive sleep apnea have a higher likelihood of developing dysbiosis (Ko et al., 2019; Neroni et al., 2021). Additionally, though not unique to individuals with PTSD, individuals with poor sleep have higher rates of impulsivity, leading to a higher consumption of ultra-processed foods or substance use (Guo et al., 2023; Whatnall et al., 2021).

Sleep quality improves with increased connectedness, increased exercise, and mindfulness practice (Sugden et al., 2024b). In addition to being the first-line therapy for insomnia (Riemann et al., 2017; Qaseem et al., 2016), cognitive behavioral therapy insomnia (CBT-i) is emerging as an effective tool for PTSD. In a randomized clinical trial, Talbot et al. showed a six-month improvement in sleep quality in 45 patients (Talbot et al., 2014). In a larger study of 110 individuals with PTSD, Pigeon et al. also showed improved sleep quality over 20 weeks (Pigeon et al., 2022). Ho et al., in their meta-analysis of 11 randomized control studies, showed that CBT-i was effective in improving PTSD-related sleep disorders, and it was a feasible treatment; nevertheless, the authors called for additional studies (Ho et al., 2016).

### 3.4 Stress management

Hypervigilance is another key feature of PTSD (Shalev et al., 2017) and is the clinical manifestation of neuroception and chronic HPA activation. In addition to the co-morbid mental health symptoms, the chronic cortisol state confers a 50% increased likelihood of developing cardiovascular disease (Cohen et al., 2015). Frontline healthcare providers who experienced trauma-related events demonstrated a higher likelihood of developing dysbiosis that persisted for over 6 months (Gao et al., 2022).

Distress tolerance is the ability to tolerate stress and negative emotions, and this skill has been at the core of psychotherapies used to help improve core symptoms. They are particularly valuable in providing a counter-narrative to the cognitive dissonance the individual oftentimes creates as a means to survive. In 2023, the revised Departments of Veterans Affairs and Defense (VA/DoD) *Clinical Practice Guideline (CPG) for Posttraumatic Stress Disorder* recommended Prolonged Exposure (PE), Cognitive Processing Therapy (CPT), and Eye Movement Desensitization and Reprocessing (EMDR) (Overview of Psychotherapy for PTSD, 2024). Additionally, Dialectical Behavioral Therapy (DBT) has shown superiority over CPT in women with complex trauma (Bohus et al., 2020). Sensorimotor therapies may also be helpful in improving dissociative or fawning symptoms commonly experienced in PTSD (Ogden and Minton, 2000; Ogden et al., 2006).

Metacognitive therapies like mindfulness, meditation, yoga, and Mindfulness-Based Cognitive Therapy (MBCT) have also been shown to improve executive cognitive decision-making (Boyd et al., 2018). These metacognitive practices decrease chronic cortisol’s effects by working in a top-down approach, increasing the function of the parietal lobe, posterior and anterior cingulate cortex, and the PFC (Garland and Howard, 2018). Additionally, mindfulness practices help improve sleep quality and can improve personal and community connections (Dahl and Davidson, 2019); however, some experts warn that those who struggle with managing distress tolerance may also struggle with developing a mindfulness practice (Follette et al., 2015). Ehlers and Clark proposed that PTSD-related symptoms develop and are maintained by the individual’s perceived levels of threat and shame (Ehlers and Clark, 2000). Conversely, self-compassion is a healthy, alternative response to trauma as it may ameliorate PTSD-related symptoms (Germer and Neff, 2015). Winders et al. showed in their review that increased self-compassion was associated with reduced trauma-related symptoms (Winders et al., 2020). Mindfulness based activities have also shown to improve stress-induced dysbiosis (Das et al., 2023).

### 3.5 Connectedness

Emotional numbing has been shown to be an early predictor of PTSD symptom severity (Feeny et al., 2000), which seems to compromise emotional development, as described by Winnicott (1958). There tends to be a steady rise in loneliness, which is an antithesis to the ability to be alone: “with cycles of reinforcement learning, individuals learn a maladaptive, distracted thinking style that uses worry to focus on the future” (Brewer and Roy, 2021). Using the construct of connectedness that was created by Gia Merlo, connectedness encompasses social connectivity, happiness, spirituality, compassion, and purpose and meaning-making (Merlo, 2024). Connectedness relies on developing a healthy relationship with self, with others, with community, and with the world (Merlo, 2024).

Loneliness is the absence of social and emotional connectivity, and although loneliness does not predispose to trauma-related symptoms, the incidence of trauma predisposes the likelihood of loneliness (Fox et al., 2021). Additionally, loneliness has also been independently associated with worsening health, poorer sleep, and a higher likelihood of developing a substance use disorder (Office of the Surgeon General (OSG), 2023a). Finally, loneliness has been shown to accelerate the aging process in people with PTSD (Palmer et al., 2022) and may contribute to deficits in working memory (Sippel et al., 2021). Conversely, social connections have been pivoting in helping the public adapt and recover from COVID-19 and have been linked to post-traumatic growth (Matos et al., 2021) and a sense of well-being (Ruppel et al., 2022).

In their systematic review, Folk and Dunn identified the practice of gratitude as one of the top strategies to achieve and maintain happiness (Folk and Dunn, 2023). Gratitude is also a predictor of well-being and has been shown to have an inverse relationship with the severity of PTSD-related symptoms (Van Dusen et al., 2015; Richardson and Gallagher, 2021).

Next, spirituality is associated with greater self-rated health, fewer health complaints, and greater life satisfaction. A higher spiritual practice increases the probability of higher life satisfaction and decreases the probability of worse health and more frequent health complaints (Dankulincova Veselska et al., 2018). The practice of spirituality has also been linked with the activation of BDNF and other neurotrophic growth factors within stress disorders like PTSD (Varghese et al., 2021).

Finally, purpose and meaning-making are the sinews that bind the factors of connectedness together. Vicktor Frankl postulated that finding purpose and meaning is essential in the recovery from trauma and is a key feature of logotherapy (Frankl, 1966). The Japanese conceptualize a life worth living as *Ikigai*, which connects life legacy, the belief that an individual’s past contributes to the present experience, and life momentum, the belief that an individual’s present experience helps achieve a desired future (Kono and Walker, 2021). After following close to 13,000 individuals in the Health and Retirement Survey, Kim et al. showed a correlation between those with a higher sense of purpose and improved sleep, improved health, and less loneliness and depressive symptoms (Kim et al., 2022). Similarly, those who maintained a purpose in life develop healthier emotional recoveries from trauma-related experiences (Schaefer et al., 2013).

### 3.6 Avoidance of toxic exposure

As previously described, PTSD and substance use share a common vulnerability and, more notably, the bidirectional relationship between trauma and substance use (María-Ríos and Morrow, 2020). Estimates suggest that more than 40% of individuals presenting for substance use treatment also meet the criteria for PTSD (Cottler et al., 1992). Experts are also noticing common behavioral personality traits among the two populations. These include a history of impulsivity, the tendency to engage in risky behavior with a lack of planning, cue reactivity, and emotional responses to neutral or salient events, and a learned fear response (Cottler et al., 1992). Additionally, shame and shame triggers are barriers to the treatment of both PTSD and substance use (Batchelder et al., 2022). Left untreated, substance use disorders lead to potentially worsening neuroinflammation and disease outcomes (Salloum and Thase, 2000; Kip and Parr-Brownlie, 2023). Chronic substance use has also been shown to affect the gut microbiota, leading to dysbiosis (Salavrakos et al., 2021; Meckel and Kiraly, 2019).

Prolonged exposure (PE), a type of cognitive behavioral therapy, focuses on confronting trauma-related memories, feelings, and situations the individual has been avoiding. Typically, sessions are weekly and last 8–15 weeks. PE has been shown to be effective with individuals who have experienced diverse traumas (Zhou et al., 2020). Recently, Back et al. have shown in U.S. Veteran populations that PE has also been effective in combined PTSD and SUD (Back et al., 2019). On the other hand, contingency management (CM) is a treatment modality that rewards participants for abstinence from substance use, and a Pfund et al. meta-analysis notes that CM is one of the effective treatments for multiple substances (i.e., methamphetamine, opioids, alcohol) (Pfund et al., 2022). There is also a growing body of literature noting how CM may be an effective modality for SUD and PTSD (Wells et al., 2022).

## 4 Social-ecological model of trauma

One of the challenging aspects of treating individuals with trauma is trying to unwind the complexities of trauma, and the Social-Ecological Model helps restack and refocus this process to better understand the individual (Gultekin et al., 2019). The first rung is to understand the individual factors: gender, age, education, temperament, and mental health status. Next are the interpersonal factors, like family and peer groups and influence, social networks, and the family or social networks’ history of trauma. Community and organizational factors include the quality and opportunities offered in the community, support within the educational system, availability of quality food, safe areas to exercise, transportation networks, and multiple other factors that contribute to socioeconomic position (SEP). Next are the cultural and developmental norms, including individual cultural norms, cultural religious beliefs, community identities, and cognitive and maturation development. Finally, the period of time that the individual lives includes periods of war, governmental programs, and other systemic programs that may enhance and support conflicts and challenges (TIP 57, 2024; Office of the Surgeon General (OSG), 2023b).

Challenges with psychotherapy and improving connectedness involve helping individuals navigate through the potential impacts of this multilayered model. As such, within the healthcare system, due to the impact and outreach of the Social-Ecological Model of Trauma, there is a need to approach each potential patient encounter through the lens of trauma-informed care, similar to how universal precautions are used to prevent blood-borne disease (Merlo and Sugden, 2023).

## 5 Conclusion

Despite the expanding number of medications and psychotherapies, the negative impact of mental health, particularly depression and trauma-related symptoms, continues to grow, as reflected by the increase in disability-adjusted life-years (DALYs), years lived with disability (YLDs), and years of life lost (YLLs) (GBD 2019 Mental Disorders Collaborators, 2022). The impacts of mental health are projected to be one of the top health concerns by 2030 (Wu et al., 2023), and as such, lifestyle interventions have a place in the treatment of PTSD. The ongoing public mental health crisis draws attention to being able to accurately diagnose and treat trauma-induced symptoms and, when possible, provide preventative treatment. Lifestyle approaches are often preferred by patients (Richardson et al., 2024) and have been shown to improve quality of life, decrease pain, and be cost-effective compared to standard care (Eriksson et al., 2010). As such, lifestyle interventions are well-positioned to help shift healthcare to a preventative model and to reduce inequities (Jain et al., 2023).

Trauma can be seen in multiple levels, including prodromal, symptomatic, and in some cases, complex trauma involving multiple neuronal pathways. Much has developed in the last 30 years in the trauma literature, including the construct of the window of tolerance (Siegel, 1999), the role of social and ecological factors (Gultekin et al., 2019), and the impact of comorbid substance use (María-Ríos and Morrow, 2020). Further research is still needed, including the emerging role of the trillions of microbes within the gut and their continued impact on the brain via the brain gut microbiota system (Wargo, 2020). Given the shared enteric embryonic origins between the gut and brain and the extensive neuronal communication pathways between the brain and gut (Sharkey and Mawe, 2023), understanding the impact of trillions of microbes may hold the future for the next-generational treatments (Crowley et al., 2019).

Indeed, lifestyle psychiatry plays an important role in this conversation. The six pillars have an important role in gut health, neuroinflammation, and PTSD symptom relief. Historically, healthcare professionals receive limited evidence-based nutrition and lifestyle intervention instruction during their formal medical education (Marx et al., 2023). We encourage medical organizations to follow the Royal Australian and New Zealand College of Psychiatrists and the World Federation of Society for Biological Psychiatry to adopt lifestyle guidelines within their mental health recommendations (Merlo and Sugden, 2024). Refreshingly, the American Psychiatric Association is actively developing educational material for mental health practitioners at all levels of training (Merlo and Sugden, 2024).
